# Differential correlation network analysis identified novel metabolomics signatures for non-responders to total joint replacement in primary osteoarthritis patients

**DOI:** 10.1007/s11306-020-01683-1

**Published:** 2020-04-25

**Authors:** Christie A. Costello, Ting Hu, Ming Liu, Weidong Zhang, Andrew Furey, Zhaozhi Fan, Proton Rahman, Edward W. Randell, Guangju Zhai

**Affiliations:** 1 Discipline of Genetics, Faculty of Medicine, Memorial University of Newfoundland, St. John’s, NL A1B 3V6 Canada; 2 Department of Computer Science, Faculty of Science, Memorial University of Newfoundland, St. John’s, NL Canada; 3 School of Pharmaceutical Sciences, Jilin University, Changchun, People’s Republic of China; 4 Division of Orthopaedic Surgery, Faculty of Medicine, Memorial University of Newfoundland, St. John’s, NL Canada; 5 Department of Mathematics and Statistics, Faculty of Science, Memorial University of Newfoundland, St. John’s, NL Canada; 6 Discipline of Medicine, Faculty of Medicine, Memorial University of Newfoundland, St. John’s, NL Canada; 7 Discipline of Laboratory Medicine, Faculty of Medicine, Memorial University of Newfoundland, St. John’s, NL Canada

**Keywords:** Osteoarthritis, Total joint replacement, Biomarkers, Metabolomics, Network analysis

## Abstract

**Introduction:**

Up to one third of total joint replacement patients (TJR) experience poor surgical outcome.

**Objectives:**

To identify metabolomic signatures for non-responders to TJR in primary osteoarthritis (OA) patients.

**Methods:**

A newly developed differential correlation network analysis method was applied to our previously published metabolomic dataset to identify metabolomic network signatures for non-responders to TJR.

**Results:**

Differential correlation networks involving 12 metabolites and 23 metabolites were identified for pain non-responders and function non-responders, respectively.

**Conclusion:**

The differential networks suggest that inflammation, muscle breakdown, wound healing, and metabolic syndrome may all play roles in TJR response, warranting further investigation.

**Electronic supplementary material:**

The online version of this article (10.1007/s11306-020-01683-1) contains supplementary material, which is available to authorized users.

## Introduction

While total joint replacement (TJR) is the most effective intervention for advanced osteoarthritis (OA), up to one third of patients undergoing total knee replacement (TKR) and total hip replacement (THR) reported unfavourable long-term pain outcomes (Beswick et al. 2012). Such patients are classified as non-responders to TJR; our previous study (Costello et al. 2019) further subdivided non-responders to clarify whether long-term outcome from surgery sees a lack of improvement in joint pain and/or joint function. It is prudent, therefore, to identify factors that are associated with these non-responders, provide insights into the potential mechanisms leading to poor TJR outcome, and develop strategies for improving patient-reported outcome.

Using a metabolomics approach, we previously identified two metabolite ratios (acetylcarnitine (C2) to phosphatidylcholine (PC) acyl-alkyl (ae) C40:1 and PC diacyl (aa) C36:4 to isoleucine) associated with pain non-responders and one metabolite ratio (glutamine to isoleucine) associated with function non-responders (Costello et al. 2019) in plasma. However, as the previous analysis used an individual metabolite-based analytic method with a conservative significance level, we might miss other important markers that play a role in TJR response. Metabolomics has previously been used to show distinct metabolic phenotypes of OA patients (Carlson et al. 2019) and thus offers a valuable technique to identify non-responders, especially when used with a network approach. Network science is a powerful tool to investigate the relationships and interaction patterns of a set of entities and has seen many applications to biomedical research as these types of analyses do not require a priori hypotheses and can thus be used to identify data-driven subgroups in complex disease that otherwise may not have been considered due to the large number of factors often considered in these types of analyses and lack of known relationship between factors. Such data driven methods have been applied to OA research by different research groups (Hu et al. 2018; Nelson et al. 2019). In this study, we applied a differential correlation network analysis method (Hu et al. 2018) to the same dataset of our previous study (Costello et al. 2019) to identify further metabolic markers and pathways for non-responders to TJR.

## Methods

### Study participants

Participants were recruited from those undergoing TJR in St. John’s, Newfoundland and Labrador (NL), Canada (Costello et al. 2019). Diagnosis was made as per the American College of Rheumatology clinical diagnostic criteria (Altman et al. 1991) and confirmed using pathology reports post-surgery. Ethics approval for the study was received from the Health Research Ethics Authority of NL (11.311). Written consent was obtained from all study participants.

### Data collection and minimal clinically important difference (MCID)

The Western Ontario and McMaster Universities Osteoarthritis Index (WOMAC) Likert v3.0 was used to evaluate patient-reported pain and function levels of OA-affected joints. The WOMAC pain and joint function subscales score 0–20 and 0–68 respectively, with 0 representing no pain or functional difficulties. Pain non-responders were classified as a pre- to post-surgery change score less than 7 in the pain subscale and function non-responders were classified as a change score less than 22 in the joint function subscale, consistent with previously used MCID definitions (Chesworth et al. 2008; Davis et al. 2012).

### Metabolic profiling and statistical methods

Metabolic profiling was performed on plasma as per our previous study (Costello et al. 2019) using a commercially available generic metabolomic assay kit—the Biocrates AbsoluteIDQ p180 commercial kit (BIOCRATES Life Sciences AG, Innsbruck, Austria) which assesses 186 metabolites, including acylcarnitines, amino acids, biogenic amines, glycerophospholipids, monosaccharides, and sphingolipids. The full list of the metabolites is provided in Supplementary Table 1.

An in-house reproducibility assay was performed using 23 samples as reported previously (Zhai et al. 2016); the mean coefficient of variation (CV) for all metabolites was 0.07 ± 0.05 μM. Ninety percent of metabolites had a CV less than 0.10. This kit has previously been used in more than 800 studies (https://biocrates.com/literature/), and details have been described previously (Zhai et al. 2016). Briefly, profiling was completed on an API4000 Qtrap® tandem mass spectrometer with electrospray ionization (Applied Biosystems/MDS Analytical Technologies, Foster City, CA) equipped with Agilent 1100 HPLC system at the Metabolomics Innovation Centre (https://www.metabolomicscentre.ca). Amino acids and biogenic amines were separated using an Agilent reversed-phase Zorbax Eclipse XDB C18 column (3.0 mm × 100 mm; 3.5 µm particle size; 80 Å pore size) prior to injection and were analyzed in positive multiple reaction monitoring (MRM) mode. All remaining metabolites were analyzed using flow injection analysis (FIA) in positive MRM mode with the exception of glucose, which was analyzed using FIA in negative MRM mode in a subsequent injection. Data analysis of compounds injected using FIA was automated using Biocrates MetIDQ software; for compounds injected from HPLC, initial analysis was performed using Analyst 1.6.2 (SCIEX, Framingham, MA) before being imported into MetIDQ. The resulting raw metabolomics data underwent strict quality control (QC) procedures prior to analysis. Metabolites were excluded completely from analysis if more than 10% of values were below the limit of detection. Missing values for remaining metabolites were imputed using the mean value across all samples for the given metabolite. In total, 131 metabolites of 186 passed QC checks and were included in the network analysis.

Prior to network analysis, metabolite values were natural log transformed and then standardized using a Z-score. Subsequently, outliers (± 3 standard deviations away from the mean) were excluded.

### Differential correlation network analysis

We previously developed a differential correlation network algorithm where we first computed the correlations of metabolite pairs in separate phenotypically distinguished groups and then computed the differential correlations through subtraction (Hu et al. 2016). This method allows us to identify metabolite pairs that are differentially correlated in distinct phenotype groups. We applied this method to the current study, with modifications to the Z-score normalization (Eq. 1) to account for the difference in sample size between responders and non-responders.

$${r}_{diff}(i,j)=\frac{({z}_{case}-{z}_{control})}{\sqrt{\frac{1}{{n}_{case}-3}+\frac{1}{{n}_{control}-3}}}$$

where *z* is the Fisher’s *z*-transformation of correlation coefficient *r.*

$${z}_{case}\left(i,j\right)= \frac{1}{2}\mathrm{ln}\left[\frac{1+{r}_{case}(i,j)}{1-{r}_{case}(i,j)}\right], {z}_{control}\left(i,j\right)=\frac{1}{2}\mathrm{ln}\left[\frac{1+{r}_{control}(i,j)}{1-{r}_{control}(i,j)}\right]$$

The significance level of a computed differential correlation for a metabolite pair was assessed using permutation testing, where phenotype labels were randomly shuffled multiple times to create a null hypothesis that there was no association between the metabolite concentrations and the phenotype status. For this study, we performed a 1000-fold permutation testing and set the significance level threshold to p < 0.01.

## Results

### Descriptive statistics

A total of 704 patients with available baseline plasma samples were included. Patients were excluded from further analysis due to missing WOMAC data (n = 188), non-primary OA (n = 48) and WOMAC pain and function baseline scores less than 7 and 22 respectively (n = 7), as per our previous study (Costello et al. 2019), and 461 patients remained for final analysis. In total, 15.1% of patients (n = 67/445) were classified as pain non-responders, while 16.0% of patients (n = 73/455) were classified as function non-responders. 72.4% of patients (n = 318/439) underwent TKR; 27.6% of patients underwent THR (n = 121/439). There was no significant difference in the prevalence of non-responders between TKR and THR (p = 0.80). Supplementary Table 2, which was presented in our previous study (Costello et al. 2019), shows the characteristics of the study cohort along with the associations between non-responders and epidemiological factors.

### Pain non-responders

**Fig. 1:**
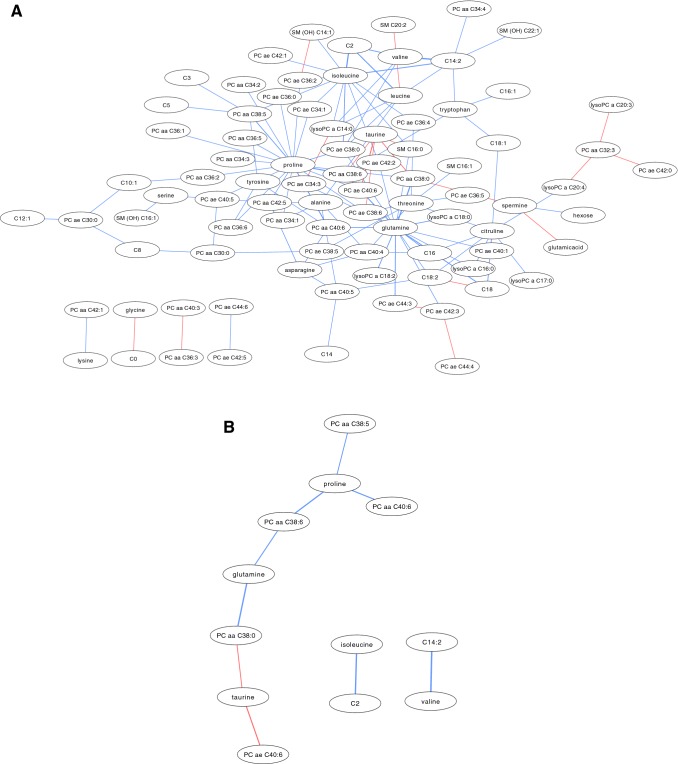
**a** Differentially correlated metabolite network of pain non-responders (p < 0.05). (Red indicates positive differential correlation; blue indicates negative differential correlation). **b** Differentially correlated metabolite network of pain non-responders (p < 0.01). (Red indicates positive differential correlation; blue indicates negative differential correlation). (PC = phosphatidylcholine; aa = diacyl; ae = acyl alkyl; C2 = acetylcarnitine, C14:2 = tetradecadienylcarnitine) (Color figure online)

At significance level p < 0.05, over 100 differential metabolite correlations were found in pain non-responders (Fig. 1a). At significance level p < 0.01, 12 metabolites were correlated differently between pain non-responders and responders; eight in a central network (encompassing the largest number of significantly correlated metabolites) and two separate pairs of correlated metabolites (Fig. 1b). These networks included five PCs, four amino acids, two acylcarnitines, and one biogenic amine. Proline was the most connected node, with three correlated edges. All metabolites are positively correlated, indicating significantly more highly positive correlations for these metabolites in non-responders than responders, except the correlations of taurine with two PCs (PC aa C38:0 and PC ae C40:6), indicating significantly less highly positive correlations for these metabolites in non-responders than responders.

### Function non-responders

**Fig. 2:**
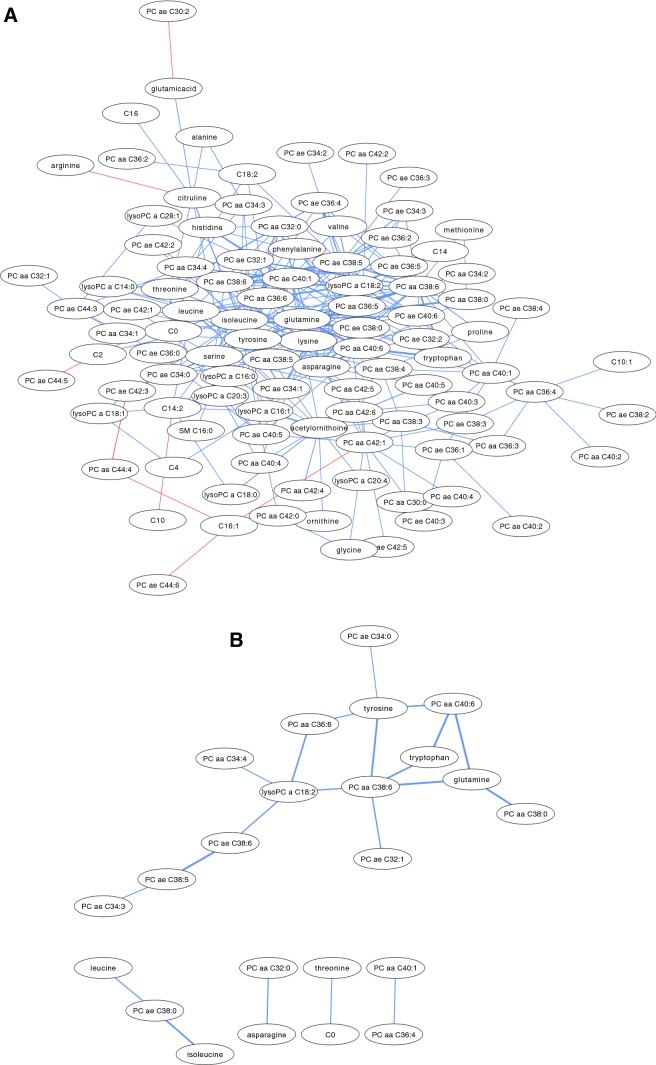
**a** Differentially correlated metabolite network of function non-responders (p < 0.05). (Red indicates positive differential correlation; blue indicates negative differential correlation). **b** Differentially correlated metabolite network of function non-responders (p < 0.01). (Red indicates positive differential correlation; blue indicates negative differential correlation). (PC = phosphatidylcholine; lysoPC = lysophosphatidylcholine; aa = diacyl; ae = acyl alkyl; C0 = carnitine) (Color figure online)

At significance level p < 0.05, over 250 differential metabolite correlations were found in function non-responders (Fig. 2a). At significance level p < 0.01, 23 metabolites were correlated differently between function non-responders and responders; 14 in a central network, three in a second, smaller network, and three separate pairs of correlated metabolites (Fig. 2b). These networks included 14 PCs, seven amino acids, one lysophosphatidylcholine (lysoPC), and carnitine. PC aa C36:8 was the most connected node, with five correlated edges. All metabolites were positively correlated in function non-responders, indicating that these metabolites had a significantly more positive correlation in non-responders than responders.

Five metabolites overlapped between pain and function non-responders: glutamine, isoleucine, PC aa C38:0, PC aa C38:6, and PC aa C40:6. The negatively correlated relationships with glutamine and two PCs, PC aa C38:0 and PC aa C38:6, also overlap between the two networks.

## Discussion

In total, 12 metabolites were differentially correlated in pain non-responders. The presence of isoleucine, C2, and a number of PCs in the network supports the metabolite ratio findings of our previous study (Costello et al. 2019) while having identified additional metabolites and pathways to be associated with pain non-responders. Interestingly, glutamine, PCs, and taurine all play roles in fatty acid metabolism; glutamine metabolism through the tricarboxylic acid (TCA) cycle can lead to fatty acid production and eventually PC production (Ridgway 2013). Taurine also has a known role in PC metabolism, lowering conversion of phosphatidylethanolamine to PC in membranes by inhibiting the enzyme which catalyzes this conversion (Schaffer et al. 2010). Fatty acid and PC synthesis are both tightly linked to metabolic syndrome (Wakil and Abu-Elheiga 2009), which is commonly associated with OA (Courties et al. 2017). The most connected metabolite in the network, proline, is especially abundant in collagen, an integral protein in both bones and cartilage. Proline supplementation with arginine, a proline precursor, has been shown to positively influence wound healing (Raynaud-Simon et al. 2012), an important factor following a surgery such as TJR. Additionally, glutamine also plays an important role in increased wound healing (Guo and Dipietro 2010). Together, proline and glutamine indicate a possible role for wound healing in lack of pain improvement following TJR.

In total, 23 metabolites were differentially correlated in function non-responders. The presence of glutamine and isoleucine in the network also supports the findings of our previous study (Costello et al. 2019) while adding additional metabolites and pathways to be associated with function non-responders. The elevated ratio of lysoPCs to PCs has previously been associated with advanced knee OA and could predict the risk of TJR in 10 year follow-up (Zhang et al. 2016). This ratio is also associated with knee OA progression measured by the cartilage volume loss over 2 years on MRI (Zhai et al. 2019a, b). The presence of a lysoPC negatively correlated with a number of PCs indicates that an alteration of the PC to lysoPC conversion, and thus an increase of downstream inflammatory mediators produced through this conversion, could be associated with function non-responders. Carnitine, another metabolite in this network, has also previously been associated with OA; two separate studies have shown decreased serum levels of acylcarnitines to be associated with OA patients, potentially due to impairment of chondrocyte repair (Zhai 2019). A connection has also previously been established between threonine and carnitine; in a rat model fed with a diet deficient in lysine and threonine, skeletal muscles became deficient in carnitine (Khan and Bamji 1979). Among the symptoms of carnitine deficiency is muscle necrosis; loss of muscle, leading to prosthesis instability, could influence the lack of improvement in function non-responders following TJR.

Interestingly, some metabolites and correlated relationships overlapped between pain and function non-responders. Isoleucine, a branched chain amino acid (BCAA), was present in both networks. BCAAs have been found to be associated with knee OA in two independent cohorts (Zhai et al. 2018), suggesting the importance of further investigations of potential connections between OA and these amino acids, especially in the case of non-responders. The negative correlation between glutamine and PCs also overlapped between these two networks. Glutamine plays a role in a number of metabolic pathways and glutaminolysis can eventually lead to fatty acid production through a number of pathways including the TCA cycle (Curi et al. 2017), which has been implicated to be altered in OA (Zhai 2019).

In conclusion, the increased presence of both PCs and BCAAs in networks for pain and function non-responders strengthens a potential connection between inflammation and muscle breakdown in non-responders to TJR. Furthermore, new correlated metabolites highlight potential roles for metabolic syndrome and wound healing in pain non-responders to OA, warranting further investigation into potential metabolic alterations in these pathways and their role in clinical improvement following TJR.

## Electronic supplementary material

Below is the link to the electronic supplementary material.

Supplementary file1 (DOCX 14 kb)

Supplementary file2 (DOCX 16 kb)

## Data Availability

Research data is available by request subject to ethical approval.
