# Supplementary material for: Differential correlation network analysis identified novel metabolomics signatures for non-responders to total joint replacement in primary osteoarthritis patients
Source: Metabolomics. 2020 Apr 25;16(5):61. doi: 10.1007/s11306-020-01683-1 (PMC7183485; doi:10.1007/s11306-020-01683-1)
Supplement: Supplementary file 1 — Supplementary file1 (DOCX 14 kb) [file 11306_2020_1683_MOESM1_ESM.docx]

**Supplementary Table 1. Biocrates AbsoluteIDQ p180 Full Metabolite List**

| **Metabolite Class** | **Number** | **Metabolite Name or Abbreviation** | **Biological Relevance (Selected Examples)** |
| --- | --- | --- | --- |
| Amino acids | 21 | Alanine, arginine, asparagine, aspartate, citrulline, glutamine, glutamate, glycine, histidine, isoleucine, leucine, lysine, methionine, ornithine, phenylalanine, proline, serine, threonine, tryptophan, tyrosine, valine | Amino acid metabolism, urea-cycle, activity of gluconeogenesis and glycolysis, insulin sensitivity, neurotransmitter metabolism, oxidative stress |
| Carnitine | 1 | C0 | Energy metabolism, fatty acid transport and mitochondrial fatty acid oxidation, ketosis, oxidative stress, mitochondrial membrane damage |
| Acylcarnitine | 25 | C2, C3, C3:1, C4, C4:1, C5, C5:1, C6(or C4:1-DC), C6:1, C8, C9, C10, C10:1, C10:2, C12, C12:1, C14, C14:1, C14:2, C16, C16:1, C16:2, C18, C18:1, C18:2 |  |
| Hydroxy- and dicarboxyacylcarnitines | 14 | C3-OH, C4-OH(or C3-DC), C5:1-DC, C5-DC(or C6-OH), C5-M-DC, C5-OH(or C3-DC-M), C7-DC, C12-DC, C14:1-OH, C14:2-OH, C16:1-OH, C16:2-OH, C16-OH, C18:1-OH |  |
| Biogenic amines | 19 | Acetylornithine, asymmetric dimethylarginine, total dimethylarginine, alpha-Aminoadipic acid, carnosine, creatinine, histamine, kynurenine, methioninesulfoxide, nitrotyrosine,hydroxyproline, phenylethylamine, putrescine, sarcosine, serotonin, spermidine, spermine, taurine |  |
| Lyso-phosphatidylcholines | 14 | LysoPC a C14:0/C16:0/C16:1/C17:0/C18:0/C18:1/ C18:2/ C20:3/C20:4/C26:0/C26:1/C28:0/C28:1 | Degradation of phospholipids, membrane damage, signalling cascades, fatty acid profile |
| Diacyl-phosphatidylcholines | 38 | PC aa C24:0/C26:0/C28:1/C30:0/C30:2/C32:0/ C32:1/C32:2/C32:3/C34:1/C34:2/C34:3/C34:4/ C36:0/C36:1/C36:2/C36:3/C36:4/C36:5/C36:6/ C38:0/C38:1/C38:3/C38:4/C38:5/C38:6/C40:1/ C40:2/C40:3/C40:4/C40:5/C40:6/C42:0/C42:1/ C42:2/C42:4/C42:5/C42:6 | Dyslipidaemia, membrane composition and damage, fatty acid profile, activity of desaturases |
| Acyl-alkyl- phosphatidylcholines | 38 | PC ae C30:0/C30:2/C32:1/C32:2/C34:0/C34:1/ C34:2/C34:3/C36:0/C36:1/C36:2/C36:3/C36:4/ C36:5/C38:0/C38:1/C38:2/C38:3/C38:4/C38:5/ C38:6/C40:1/C40:2/C40:3/C40:4/C40:5/C40:6/ C42:0/C42:1/C42:2/C42:3/C42:4/C42:5/C44:3/ C44:4/C44:5/C44:6 |  |
| Sphingomyelins | 10 | SM C16:0, SM C16:1, SM C18:0, SM C18:1, SM C20:2, SM C22:3, SM C24:0, SM 24:1, SM C26:0, SM C26:1 | Signalling cascades, membrane damage (eg, neurodegeneration) |
| Hydroxysphingomyelins | 5 | SM (OH) C14:1, SM (OH) C16:1, SM (OH) C22:1, SM (OH) C22:2, SM (OH) C24:1 |  |
| Monosaccharides | 1 | Hexose (H1) | Carbohydrate metabolism |
| **Total** | **186** |  |  |
