# Supplementary material for: Differential correlation network analysis identified novel metabolomics signatures for non-responders to total joint replacement in primary osteoarthritis patients
Source: Metabolomics. 2020 Apr 25;16(5):61. doi: 10.1007/s11306-020-01683-1 (PMC7183485; doi:10.1007/s11306-020-01683-1)
Supplement: Supplementary file 2 — Supplementary file2 (DOCX 16 kb) [file 11306_2020_1683_MOESM2_ESM.docx]

**Supplementary Table 2. Epidemiological Factors of Pain or Function Responders and Non-Responders**

| Variables | Responders  (n=344; 78.4%) | Non-Responders  (n=95; 21.6%) | Pain Responders (n=378; 84.9%) | Pain Non-Responders (n=67; 15.1%) | Function Responders (n=382; 84.0%) | Function Non-Responders (n=73; 16.0%) |
| --- | --- | --- | --- | --- | --- | --- |
| Age (years)^†^ | 65.3±7.8 | 64.3±7.6 | 65.2±7.8 | 64.6±7.2 | 65.6±7.7 | 63.6±7.8* |
| % Female^††^ | 58 | 56 | 59 | 55 | 56 | 58 |
| BMI (kg/m^2^)^†^ | 34.2±7.3 | 34.0±6.7 | 34.1±7.3 | 33.7±6.0 | 34.0±7.2 | 34.0±6.7 |
| Knee Replacement (%)^††^ | 72.1 | 73.7 | 72.5 | 73.1 | 70.9 | 74.0 |
| Hybrid Hip Prosthesis (%)^†††^ | 62.5 | 48.0 | 61.5 | 44.4 | 64.0 | 47.4 |
| Stryker CR Knee Prosthesis (%)^†††^ | 81.5 | 88.9 | 88.2 | 83.7 | 87.0 | 91.7 |
| Diabetes (%)^††^ | 18.3 | 22.1 | 17.8 | 25.8 | 18.4 | 22.2 |
| Revisions (%)^†††^ | 1.2 | 11.6** | 1.3 | 14.9** | 1.3 | 13.7** |
| Total Cholesterol (ln(mmol/L))^†^ | 1.53±0.24 | 1.55±0.23 | 1.53±0.24 | 1.53±0.23 | 1.53±0.24 | 1.57±0.23 |
| HDL Cholesterol (ln(mmol/L)))^†^ | 0.15±0.25 | 0.20±0.27 | 0.16±0.26 | 0.21±0.28 | 0.16±0.26 | 0.19±0.26 |
| LDL Cholesterol (ln(mmol/L)))^†^ | 0.97±0.35 | 0.98±0.34 | 0.97±0.35 | 0.94±0.35 | 0.97±0.35 | 1.01±0.35 |
| Non-HDL Cholesterol (ln(mmol/L)))^†^ | 1.22±0.30 | 1.23±0.30 | 1.23±0.30 | 1.19±0.31 | 1.22±0.30 | 1.26±0.30 |
| Total Cholesterol to HDL Cholesterol Ratio^†^ | 1.38±0.26 | 1.35±0.29 | 1.37±0.26 | 1.31±0.31 | 1.36±0.26 | 1.38±0.28 |
| Triglycerides (ln(mmol/L)))^†^ | 0.40±0.48 | 0.38±0.52 | 0.40±0.48 | 0.34±0.50 | 0.39±0.47 | 0.41±0.55 |
| WOMAC Pain Baseline Score^†^ | N/A | N/A | 14.7±3.4 | 12.4±4.5** | N/A | N/A |
| WOMAC Function Baseline Score^†^ | N/A | N/A | N/A | N/A | 48.0±10.2 | 44.5±12.9* |

** p<0.05 **p<0.001*

^†^t-test used in analysis ^††^Chi-squared test used in analysis ^†††^Fisher’s exact test used in analysis

(BMI = body mass index; CR = cruciate retaining; HDL = high density lipoprotein; LDL = low density lipoprotein;
WOMAC = Western Ontario and McMaster University Osteoarthritis Index)

Pain and function non-responders defined based on WOMAC absolute change score criteria (For pain non-responders, change score < 7 out of the total 20; for function non-responders, change score < 22 out of the total 68; for non-responders column, non-responders had to meet at least one of the previous two non-responder criteria)
